# Enhanced Arterial Spin Labeling Magnetic Resonance Imaging of Cerebral Blood Flow of the Anterior and Posterior Circulations in Patients With Intracranial Atherosclerotic Stenosis

**DOI:** 10.3389/fnins.2021.823876

**Published:** 2022-02-17

**Authors:** Hongwei Yu, Yangchen Li, Yibo Feng, Linwei Zhang, Zeshan Yao, Zunjing Liu, Wenwen Gao, Yue Chen, Sheng Xie

**Affiliations:** ^1^Department of Radiology, China-Japan Friendship Hospital, Beijing, China; ^2^Graduate School of Peking Union Medical College, Chinese Academy of Medical Sciences and Peking Union Medical College, Beijing, China; ^3^Department of Ultrasound Medicine, China-Japan Friendship Hospital, Beijing, China; ^4^Department of Neurology, China-Japan Friendship Hospital, Beijing, China; ^5^AnImageTech, Beijing Co., Ltd, Beijing, China

**Keywords:** arterial spin labeling, intracranial atherosclerotic stenosis, magnetic resonance imaging, ultrasonography, hemodynamics

## Abstract

**Objectives:**

This study analyzed differences in the mean cerebral blood flow (mCBF) and arterial transit time (ATT) of the anterior and posterior circulations between patients with intracranial atherosclerotic stenosis (ICAS) and control subjects. We also investigated the correlation between ATT and mCBF in the two groups, and evaluated whether the blood flow velocity of the extracranial carotid/vertebral arteries can influence mCBF.

**Methods:**

A total of 32 patients with ICAS were prospectively enrolled at the Radiology Department of the China-Japan Friendship Hospital between November 2020 and September 2021. All patients had extensive arterial stenosis, with 17 having cerebral arterial stenosis in the anterior circulation and 15 in the posterior circulation. Thirty-two healthy subjects were enrolled as a control group. Enhanced arterial spin labeling (eASL) imaging was performed using a 3.0-T GE magnetic resonance imaging scanner, and all patients underwent carotid and vertebral Doppler ultrasound examinations. CereFlow software was used for post-processing of the eASL data, to obtain cerebral perfusion parameters such as mCBF and ATT. Independent samples *t*-tests were used to analyze and compare mCBF and ATT of the anterior circulation (frontal lobe, parietal lobe, and insula) and posterior circulation (occipital lobe, cerebellum) between the patient and control groups. The relationships of ATT and mCBF in the two groups were evaluated with Pearson’s correlation. The blood flow velocity of the extracranial internal carotid/vertebral arteries, including the peak systolic velocity (PSV), end diastolic velocity (EDV), mean PSV (mPSV), and mean EDV (mEDV), was compared between the control and study groups using *t*-tests. Multiple linear regression analysis was then applied to determine the factors associated with mCBF in the two groups.

**Results:**

The mCBFs of the anterior and posterior circulations in the patient group were lower than those of the control group. The ATTs in the patient group were all significantly longer than those of the control group (*p* < 0.05). Except for the insula in the control group, significant correlations were found between ATT and mCBF in all other investigated locations in the two groups (*p* < 0.05). The blood flow velocity of the extracranial internal carotid/vertebral arteries differed significantly between the control and patient groups (*p* < 0.05). The multiple linear regression analysis revealed that in patients with ICAS, mPSV of the vertebral arteries and local ATT correlated with mCBF of the occipital lobes and the cerebellum, respectively (*p* < 0.05). In contrast, there was no significant correlation within the anterior circulation (frontal lobes, parietal lobes, and insula).

**Conclusion:**

There was a significant relationship between ATT and mCBF in patients with ICAS. Extracranial blood flow may influence intracranial hemodynamics in the posterior circulation in patients with ICAS. The maintenance of extracranial blood flow is of great significance in the preservation of intracranial hemodynamics.

## Introduction

Intracranial atherosclerotic stenosis (ICAS) is a major cause of ischemic stroke in the Chinese population, accounting for 30–50% of ischemic stroke (Wang Y. et al., 2014). The risk of hypoperfusion in patients with ICAS is an important consideration in prognosis and the development of treatment strategies. At present, the commonly used methods for perfusion evaluation are CT perfusion (CTP) imaging using an exogenous contrast agent, and magnetic resonance dynamic susceptibility contrast perfusion-weighted imaging. CTP is routinely used to evaluate cerebral hemodynamics because it provides relatively accurate perfusion quantification, including cerebral blood flow (CBF), cerebral blood volume, and mean transit time (MTT; Kang et al., 2008). However, the administration of exogenous contrast agents is invasive and presents risks from contrast agent allergy and renal interstitial fibrosis (Zaharchuk, 2007). Furthermore, the perfusion results obtained from patients with severe ICAS may present with errors because of changes in the blood–brain barrier (Leng et al., 2019). The three-dimensional pseudo-continuous arterial spin labeling (3D-pCASL) technique does not require administration of exogenous contrast agents, instead using magnetically labeled hydrogen protons in the blood as a freely diffusible contrast agent to measure CBF. It is advantageous as it is non-invasive, is repeatable, and does not require vascular injection of a contrast agent for the evaluation of cerebral blood perfusion. A previous study showed a significant correlation between perfusion data from ASL and (CTP) imaging in patients with moyamoya disease (Wang R. et al., 2014). However, the quantification of CBF by 3D-pCASL is affected by the arterial transit time (ATT). The delay in the time the hydrogen protons in the arterial blood take to flow through the labeled area to the acquisition area is likely to result in the loss of labeled signals and errors, leading to reduced CBF signals (Cheng et al., 2012; Ferré et al., 2013). Therefore, uncertainty in the ATT weakens the potential advantages of 3D-pCASL for the accurate and quantitative measurement of CBF. In contrast, the enhanced arterial spin labeling (eASL) imaging technique uses multiple post-labeling delay (PLD) times to calculate ATT (Detre and Alsop, 1999). Compared with a single PLD or three PLDs, eASL can better quantify blood perfusion and ATT, reduce physiological background noise, and improve the accuracy of mean CBF (mCBF) measurement (Wells et al., 2010; Wang et al., 2013).

Patients with ICAS usually have hemodynamic disorders, resulting in abnormal cerebral blood flow and elongation of ATT. ATT is a physiological parameter that reflects the time required for labeled spins to reach the region of interest in the brain (Wang et al., 2003). A previous study demonstrated significant correlations between ASL, ATT, and CTP MTT in moyamoya disease (Ravindra et al., 2020). In hemispheric transient ischemic attack patients, both perfusion-weighted imaging and ASL findings were more common in the symptomatic hemisphere. Agreement between neuroradiologists regarding abnormal studies was good for ASL and perfusion-weighted imaging (Zaharchuk et al., 2012). However, most studies focused on investigation of the hemodynamic changes in the anterior circulation. The posterior circulation, which is supplied by the basilar artery formed by the confluence of bilateral vertebral arteries at the level of the brainstem, has not been widely studied using ASL. The relationship between the ATT and CBF in the posterior circulation may not be as consistent as that in the anterior circulation. The upstream blood flow from the extracranial carotid and vertebral arteries may exert an effect on the blood flow downstream. If so, the blood flow velocity of the extracranial carotid/vertebral arteries should affect the intracranial hemodynamics. In this study, we aimed to investigate the relationship between ATT and CBF in both the anterior and posterior circulation, determining the influence of extracranial flow on the intracranial hemodynamics.

## Materials and Methods

### Subjects

A total of 32 patients [15 men and 17 women, mean age (standard deviation) 66.5 ± 9.8 years] with ICAS were prospectively enrolled at the Radiology Department of the China-Japan Friendship Hospital between November 2020 and September 2021. All patients had extensive arterial stenosis, with 17 having severe cerebral arterial stenosis in the anterior circulation and 15 in the posterior circulation. The inclusion criteria were as follows: patients were diagnosed with intracranial artery stenosis by CT angiography (CTA) and magnetic resonance angiography (MRA) of the head and neck; CTA of the head and neck and carotid Doppler ultrasound (CDU) showed no severe stenosis (stenosis >50% of the normal vessel diameter) or occlusion of the extracranial carotid and vertebral arteries; and the intervals between CTA, MRA, and CDU examinations were less than 72 h. The exclusion criteria were as follows: other diseases that could affect the hemodynamics, such as intracranial space-occupying lesions, arteriovenous fistula, moyamoya disease, cerebral edema, and massive cerebral infarction; systemic diseases that could affect cerebral blood flow, such as heart failure, serious cardiovascular disease, severe anemia, and hypovolemia; and intake of medications that could markedly affect the brain blood flow. Additionally, thirty-two healthy volunteers [16 men and 16 women; mean age (standard deviation) 64 ± 1.78 years] were enrolled as the control group. The study protocol was approved by the Institutional Review Boards of the China-Japan Friendship Hospital (approval number: 2015-23). Informed consent was obtained from each subject.

### MRI Data Acquisition and Processing

A 3.0-T magnetic resonance imaging scanner (Discovery MR750, GE Healthcare, Waukesha, WI, United States) with an 8-channel head coil was used for imaging. The MRI sequences included T1-weighted imaging, T2-weighted imaging, diffusion-weighted imaging, three-dimensional time-of-flight MRA, and the eASL sequence. The perfusion-weighted images were acquired at seven consecutive pulsed pCASL labeling durations of 0.22, 0.26, 0.30, 0.37, 0.48, 0.68, and 1.18 s, with PLDs of 1.00, 1.22, 1.48, 1.78, 2.15, 2.62, and 3.32 s in the eASL sequence. The scan parameters included a 512 × 512 matrix, a 22 cm × 22 cm field of view, a 62.5-kHz bandwidth, 4-mm slice thickness, 36 slices, an echo time of 10.5 ms, and a repetition time of 5,936 ms. IDL-based RWCON code was used to reconstruct images, which were stored in the database as DICOM images.

CereFlow software (Translational MRI, LLC, Los Angeles, CA, United States) was used to process the eASL data according to the following steps (van der Thiel et al., 2018): (1) The eASL raw data and CBF data were converted into parametric cerebral perfusion maps to obtain mCBF and ATT. (2) The parametric cerebral perfusion maps were normalized into the standard brain space of the Montreal Neurological Institute (MNI), resampled after normalization, and spatially smoothed. (3) The atlas of arterial blood supply territories, ASPECTS brain atlas, and the automated anatomical labeling atlas were overlaid. (4) The mean perfusion values of each territory volume were obtained.

The perfusion regions of interest (ROIs) of the anterior circulation included the frontal lobe, parietal lobe, and insula, while the perfusion ROIs of the posterior circulation included the occipital lobe and cerebellum. Because the temporal lobe is supplied by both the anterior and posterior circulations, it was excluded from the measurements to avoid confusion.

### CT Angiography Examination

A Gemstone Spectral Imaging CT scanner (Discovery CT750 HD, GE Healthcare, United States) was used to perform CTA of the head and neck. A 50-ml dose of non-ionic contrast agent was injected into the median cubital vein in the forearm of the patient using a high-pressure syringe at a rate of 4 ml/s. The scanning was performed in the caudocranial direction, from the aortic arch to the top of the skull.

### Carotid Doppler Ultrasound Examination

A GE Logic E9 ultrasound machine (GE Healthcare, United States) with a 9L linear array probe at a frequency of 8–9 MHz was used for the CDU examinations. Bilateral common carotid, internal carotid, external carotid, and vertebral and subclavian arteries were measured conventionally by one experienced doctor who was blind to the clinical status of the subjects. The measurements of extracranial artery flow velocity were performed at the same location for each subject. The inner diameter, peak systolic velocity (PSV), end diastolic velocity (EDV), and vascular resistive index of the extracranial internal carotid and vertebral arteries were measured twice and average values were recorded.

### Evaluation of Intracranial Perfusion

The raw eASL data were post-processed using CereFlow software to generate ATT and mCBF maps of the whole brain. Based on a vessel territory atlas, the ATT and mCBF maps of the bilateral frontal lobes, parietal lobes, insula, occipital lobes, and cerebellum were generated automatically for statistical analysis.

### Statistical Analysis

Statistical analysis was performed using SPSS 25.0 software. A *p*-value < 0.05 was considered statistically significant. The continuous variables in this study followed a normal distribution and homogeneity of variance. The independent samples *t*-test was used to analyze and compare the mCBF and ATT of the anterior circulation (bilateral frontal lobes, parietal lobes, and insula) and posterior circulation (occipital lobes and cerebellum) between the patient and control groups. Pearson correlation analysis was used to evaluate the relationship between ATT and mCBF in the two groups. Differences in the blood flow velocity of the extracranial internal carotid/vertebral arteries between the two groups were compared using independent samples *t*-tests. Using PSV, EDV, and ATT as independent factors, multiple linear regression analysis was then applied to analyze the factors affecting the mCBF of the ROIs in the two groups. Because the posterior circulation is supplied by the basilar artery formed by the confluence of the bilateral vertebral arteries, the bilateral PSV, EDV, ATT, and mCBF values of the occipital lobes and cerebellum were averaged for the regression analysis.

## Results

### Comparison of Cerebral Perfusion Parameters Between the Study and Control Groups

**FIGURE 1:**
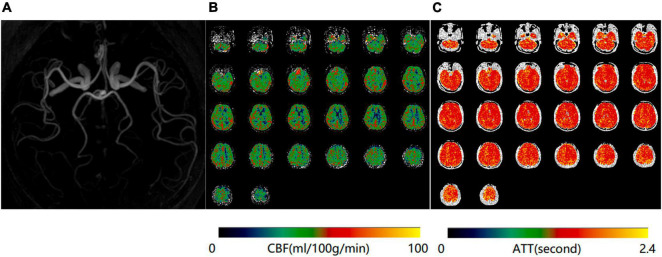
Normal magnetic resonance angiography (MRA) **(A)**, mean cerebral blood flow (mCBF) **(B)**, and arterial transit time (ATT) **(C)** maps of a 65-year-old woman in the control group.

**FIGURE 2:**
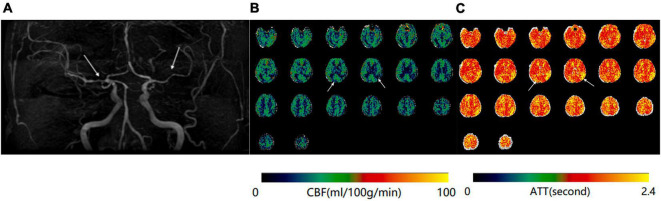
**(A)** Magnetic resonance angiography (MRA) of a 68-year-old woman shows moderate stenosis of bilateral middle cerebral arteries. **(B)** Mean cerebral blood flow (mCBF) demonstrates decreased blood perfusion in bilateral temporal and parietal lobes. **(C)** Prolongation of arterial transit time (ATT) is demonstrated at the same anatomical regions on the ATT map.

**FIGURE 3:**
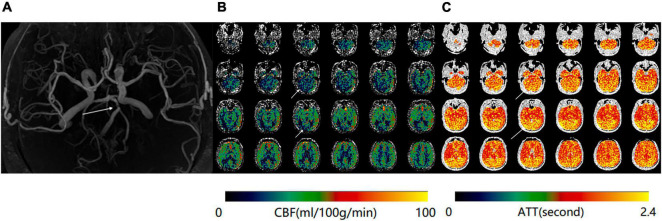
**(A)** Magnetic resonance angiography (MRA) of a 70-year-old man shows severe stenosis of the basilar artery. **(B)** Maps of mean cerebral blood flow (mCBF) demonstrate markedly decreased blood flow in the bilateral occipital lobes and cerebellum. **(C)** Arterial transit time (ATT) maps shows increased signal at the same anatomical regions.

Compared with those in the control group, the mCBF measurements in the anterior circulation (bilateral frontal lobes, parietal lobes, and insula) and posterior circulation (bilateral occipital lobes and cerebellum) were all significantly lower in the patient group. The ATTs of the ROIs were significantly longer in the patient group than in the control group (Table 1 and Figures 1–3). There were also statistically significant differences in the blood flow velocity of the extracranial internal carotid/vertebral arteries between the control and patient groups (*p* < 0.05) (Table 2).

**TABLE 1:** Comparison of the mCBF and ATT of all parts between the study and control groups.

Variables	Study group (*n* = 32)	Control group (*n* = 32)	*p*-value
Left frontal lobe mCBF (ml/100 g/min)	29.7775 ± 8.2272	40.2331 ± 5.6562	<0.001
Right frontal lobe mCBF (ml/100 g/min)	29.8481 ± 7.6619	39.0094 ± 5.6092	<0.001
Left parietal lobe mCBF (ml/100 g/min)	27.9381 ± 9.1233	39.7225 ± 5.8357	<0.001
Right parietal lobe mCBF (ml/100 g/min)	27.3203 ± 7.7837	37.7647 ± 5.8109	<0.001
Left insula mCBF (ml/100 g/min)	31.6787 ± 9.9949	39.2906 ± 4.9793	<0.001
Right insula mCBF (ml/100 g/min)	30.8713 ± 8.9645	39.9406 ± 5.8151	<0.001
Left occipital lobe mCBF (ml/100 g/min)	29.2641 ± 10.1001	39.9081 ± 7.8628	<0.001
Right occipital lobe mCBF (ml/100 g/min)	27.9938 ± 8.5938	39.0856 ± 6.5587	<0.001
Left cerebellum mCBF (ml/100 g/min)	27.4838 ± 8.8289	36.3209 ± 7.9350	<0.001
Right cerebellum mCBF (ml/100 g/min)	27.2756 ± 8.8254	36.2350 ± 7.4646	<0.001
Left frontal lobe ATT (s)	1.7400 ± 0.1471	1.6328 ± 0.0927	<0.001
Right frontal lobe ATT (s)	1.7747 ± 0.1438	1.6247 ± 0.1080	<0.001
Left parietal lobe ATT (s)	1.8112 ± 0.1596	1.6731 ± 0.1068	<0.001
Right parietal lobe ATT (s)	1.8000 ± 0.1717	1.6763 ± 0.1318	<0.001
Left insula ATT (s)	1.6700 ± 0.1204	1.5906 ± 0.1016	<0.001
Right insula ATT (s)	1.6650 ± 0.1429	1.5794 ± 0.0583	<0.001
Left occipital lobe ATT (s)	1.8569 ± 0.1391	1.6850 ± 0.1529	<0.001
Right occipital lobe ATT (s)	1.8509 ± 0.1477	1.6888 ± 0.1311	<0.001
Left cerebellum ATT (s)	1.7966 ± 0.1311	1.6591 ± 0.1471	<0.001
Right cerebellum ATT (s)	1.8278 ± 0.1267	1.6906 ± 0.1325	<0.001

*ATT, arterial transit time; mCBF, mean cerebral blood flow; s, seconds.*

**TABLE 2:** Comparison of the blood flow velocity of the extracranial internal carotid and vertebral arteries between the study and control groups.

Variables	Study group (*n* = 32)	Control group (*n* = 32)	*p*-value
PSV-LICA (cm/s)	61.5625 ± 16.2360	75.5938 ± 18.1745	0.002
EDV-LICA (cm/s)	25.1562 ± 9.2007	30.2500 ± 9.1087	0.030
PSV-RICA (cm/s)	67.7500 ± 21.6661	82.8750 ± 19.4932	0.005
EDV-RICA (cm/s)	23.6250 ± 7.4042	31.0312 ± 9.6067	<0.001
mPSV-VA (cm/s)	45.9531 ± 8.8976	50.9062 ± 8.5388	0.027
mEDV-VA (cm/s)	14.5625 ± 3.6781	16.7656 ± 4.2445	0.030

*PSV, peak systolic velocity; EDV, end-diastolic velocity; mPSV, mean peak systolic velocity; mEDV, mean end-diastolic velocity; LICA, left lateral internal carotid artery; RICA, right lateral internal carotid artery; VA, vertebral artery.*

### Correlation of Arterial Transit Time and Mean Cerebral Blood Flow

Taking the bilateral perfusion data as a whole, significant negative correlations between ATT and mCBF were found in the frontal lobes, parietal lobes, insula, occipital lobes, and cerebellum in the patient group (*r* = −0.386, *p* = 0.002; *r* = −0.386, *p* = 0.002; *r* = −0.267, *p* = 0.033; *r* = −0.573, *p* < 0.001; *r* = −0.489, *p* < 0.001, respectively).

Significant correlations between ATT and mCBF were also demonstrated in the control group, except for the insula, where the correlation did not quite reach significance (*r* = −0.239, *p* = 0.057).

### Results of the Multiple Linear Regression Analysis

There was a relationship between ATT and mCBF in the ROIs of the frontal lobes and parietal lobes in the controls, and a significant but weak effect of PSV on mCBF in the insula (β = 0.093, *p* = 0.013), but no effect of extracranial flow velocity was observed in the posterior circulation (Table 3). No significant correlations with factors that could possibly affect mCBF in the ROIs of the anterior circulation (frontal lobes, parietal lobes, and insula) were found in the patients with ICAS (*p* > 0.05) (Table 4). For the posterior circulation, the mean PSV of the vertebral arteries and ATT of the cerebellum showed a relationship with mCBF of the cerebellum (β = 0.397, *p* = 0.042; β = −0.528, *p* = 0.002, respectively). In addition, the mCBF of the occipital lobes correlated with ATT and mean PSV of the vertebral arteries (β = 0.468, *p* = 0.014; β = −0.632, *p* < 0.001, respectively) (Table 4).

**TABLE 3:** Results from multiple linear regression of the extracranial artery flow velocity, ATT on CBF in the anterior and posterior circulations of the control group.

Dependent variable	Independent variable	*B* coefficient	*p*-value
Frontal lobe mCBF	PSV-ICA	0.065 (−0.012, 0.142)	0.095
	EDV-ICA	−*0*.*074* (−*0*.*083*, *0*.*232*)	0.348
	Frontal lobe ATT	−*14*.*371* (−*27*.*736*,−*1*.*005*)	0.036
Parietal lobe mCBF	PSV-ICA	0.025 (−0.058, 0.107)	0.555
	EDV-ICA	0.105 (−0.061, 0.271)	0.21
	Parietal lobe ATT	−*14*.*230* (−*26*.*347*,−*2*.*112*)	0.022
Insula mCBF	PSV-ICA	0.093 (0.020, 0.166)	0.013
	EDV-ICA	0.055 (−0.098, 0.208)	0.476
	Insula ATT	−*9*.*059* (−*25*.*012*, *6*.*894*)	0.261
Occipital lobe mCBF	mPSA-VA	0.029 (−0.279, 0.337)	0.85
	mEDV-VA	0.686 (−0.079, 1.452)	0.077
	Occipital lobe mATT	−*15*.*104* (−*32*.*847*, *2*.*640*)	0.092
Cerebellum mCBF	mPSV-VA	0.001 (−0.344, 0.346)	0.995
	mEDV-VA	0.733 (−0.103, 1.569)	0.083
	Cerebellum mATT	−*16*.*496* (−*36*.*053*, *3*.*061*)	0.095

*ATT, arterial transit time; mPSV, mean peak systolic velocity; mEDV, mean end-diastolic velocity; mCBF, mean cerebral blood flow; PSV, peak systolic velocity; EDV, end-diastolic velocity; VA, vertebral artery; ICA, internal carotid artery.*

**TABLE 4:** Results from multiple linear regression of the extracranial artery flow velocity, ATT on CBF in the anterior and posterior circulations of the study group.

Dependent variable	Independent variable	*B* coefficient	*p*-value
Frontal lobe mCBF	PSV-ICA	0.131 (−0.017, 0.279)	0.081
	EDV-ICA	−0.053 (−0.485, 0.379)	0.807
	Frontal lobe ATT	−12.668 (−28.260, 2.924)	0.109
Parietal lobe mCBF	PSV-ICA	0.130 (−0.022, 0.282)	0.092
	EDV-ICA	0.097 (−0.337, 0.531)	0.656
	Parietal lobe ATT	−9.180 (−22.538, 4.178)	0.174
Insula mCBF	PSV-ICA	0.174 (−0.010, 0.359)	0.063
	EDV-ICA	−0.108 (−0.638, 0.421)	0.683
	Insula ATT	−11.253 (−31.067, 8.560)	0.260
Occipital lobe mCBF	mPSA-VA	0.423 (0.092, 0.754)	0.014
	mEDV-VA	−0.237 (−1.130, 0.657)	0.591
	Occipital lobe mATT	−42.319 (−63.169, −21.469)	<0.001
Cerebellum mCBF	mPSV-VA	0.334 (0.013, 0.655)	0.042
	mEDV-VA	−0.076 (−0.916, 0.764)	0.855
	Cerebellum mATT	−38.432 (−61.545, −15.318)	0.002

*ATT, arterial transit time; mPSV, mean peak systolic velocity; mEDV, mean end-diastolic velocity; mCBF, mean cerebral blood flow; PSV, peak systolic velocity; EDV, end-diastolic velocity; VA, vertebral artery; ICA, internal carotid artery.*

## Discussion

In this study, we used eASL imaging technology to analyze the perfusion state of the anterior and posterior circulations in patients with ICAS and healthy people, and investigated the relationship between ATT and mCBF in the subjects. We found that in patients with ICAS, the mCBF values of both the anterior circulation and posterior circulation were significantly lower than those of normal healthy people. Furthermore, the ATTs of the patients with ICAS were longer than those of healthy people, which is consistent with the results of previous research (Li et al., 2016; Xu et al., 2016). Stenosis of cerebral arteries caused by ICAS can result in extensive changes in cerebral blood flow distribution, which are closely related to the structure of vessel walls and the hemodynamics across the stenosis (Sangha et al., 2017; Kleindorfer et al., 2021).

We showed significant correlations between ATT and mCBF in ROIs placed in the anterior and posterior circulations in patients with ICAS, as well as in controls. As the mCBF value decreased, the ATT increased. The ATT is a measure of the time taken for the labeled spins to reach the brain tissue of an ROI, and therefore reflects both the arterial flow and intracranial arteriole flow. Because blood flow in the large arteries is very fast and takes little time to flow into the brain, most of the ATT can be attributed to the intracranial arteriole component. Some studies demonstrated the agreement of ATT and MTT in patients with vascular stenosis (Wang et al., 2003; Xu et al., 2021), and previous research suggested a feedback mechanism between the transit time and CBF to maintain stable cerebral perfusion (Haller et al., 2016; Mutsaerts et al., 2017; Cohen et al., 2020). The relationship between ATT and CBF in patients with ICAS is of great importance, especially in the posterior circulation (Jia et al., 2017; Sparaco et al., 2019). Compared with that in the arteries of the anterior circulation, the blood flow velocity in the vessels of the posterior circulation is lower, producing abnormal wall shear stress, which is a risk factor for thrombosis (Caplan, 2000; Eker et al., 2019). At the same time, atherosclerosis can lead to artery stenosis and occlusion, which slows down blood flow and results in a low perfusion state (Ya et al., 2020). In this situation, compensatory elongation of ATT will help to maintain local perfusion in a steady state. The adjustment of ATT may reflect slow collateral flow compensating for a disrupted blood supply through one of the proximal arteries (van Osch et al., 2018).

In addition to eASL, we used color Doppler ultrasound to examine the extracranial carotid/vertebral arteries and measure their blood flow velocity. Although our patients had no severe stenosis or occlusion of the extracranial carotid and vertebral arteries, there were significant differences in the blood flow velocity of the extracranial carotid and vertebral arteries between the controls and patients. In the patients with ICAS, the mean PSV of the vertebral arteries and local ATT demonstrated a significant association on the mCBF of the occipital lobes and cerebellum, but this phenomenon was not observed in the controls. We suggest that mCBF of the posterior circulation in patients with ICAS is sensitive to the change in the upstream blood supply. Under normal physiological conditions, intracranial hemodynamic mechanisms can well-adjust to pressure and blood flow changes from the extracranial internal carotid and vertebral arteries (McBryde et al., 2017). In contrast, patients with ICAS seem to have a declined ability to cope with the reduced blood inflow, suggesting a vulnerability to the decrease in perfusion (Nixon et al., 2010). We note that the association of the extracranial flow on the mCBF in patients with ICAS was demonstrated exclusively in the posterior circulation. This may be because posterior circulation vessels such as the basilar artery have less sympathetic nerve innervation, which leads to poor vascular regulation (Tissir and Goffinet, 2013). Our study supports the idea that maintenance of extracranial blood flow is of great significance in the preservation of intracranial hemodynamics, especially those of the posterior circulation.

Previous studies mostly used arterial spin labeling technology with a single PLD or three PLDs, but the mismatch between PLD and ATT among individuals is the main reason for inaccurate measurement of CBF (MacIntosh et al., 2010). In this study, eASL based on Hadamard matrix time coding was applied, and the total labeling time was divided into seven short labeling time blocks, which significantly shortened the total time for collecting multiple PLDs and allowed ATT and mCBF to be obtained at the same time (Dai et al., 2013). This sequence also facilitated quick measurement of ATT by low-resolution time coding technology, and choosing of a reasonable PLD based on this known ATT, thereby allowing a more accurate mCBF measurement to be obtained (Teeuwisse et al., 2014; von Samson-Himmelstjerna et al., 2016). CBF measurements using pCASL with multiple post-label delay acquisitions correlated well with quantitative CBF values derived from ^15^O-H_2_O PET in patients with chronic occlusive cerebrovascular disease (Kamano et al., 2013). The disadvantages of eASL lie in the longer scanning time and low signal-to-noise ratio due to the short label durations (Haller et al., 2016). Optimization of the number of post-label delay acquisitions and excitations to reduce the acquisition time with a sparse model-based image reconstruction (Tsujikawa et al., 2016), and/or use of more complex but efficient Hadamard time-encoding strategies (Amemiya et al., 2021), will be needed to establish guidelines for routine use in future clinical applications.

There are several limitations to this study. First, not all brain regions were included in the analysis, because some brain regions like the thalamus and temporal lobes are supplied by both the anterior and posterior circulation, which may cause confusion when analyzing the perfusion state. Second, the relationship between the stenoses and eASL parameters was not studied. Because some patients had extensive intracranial atherosclerotic lesions, we could not simply classify the vessel territories according to the stenosis of arteries. Sometimes, a vessel territory with a relatively mild stenosis may be ischemic because of the intracerebral steal phenomenon. Third, the CDU examinations were conducted by only one experienced doctor who was blind to the clinical status of the subjects, and we therefore had no measure of the reliability of the ultrasonographic measurements. Finally, the sample size of this study was small, and the influences of age, blood pressure, and vessel diameter on the blood flow of the cervical and vertebral arteries were not analyzed.

Overall, we used eASL to demonstrate changes in ATT and mCBF in patients with ICAS. We found that ATT correlated with mCBF in the healthy controls, as well as in the patients. Furthermore, we found that extracranial blood flow may influence intracranial hemodynamics in the posterior circulation of the patients, indicating the importance of the maintenance of upstream blood flow.

## Data Availability Statement

The raw data supporting the conclusions of this article will be made available by the authors, without undue reservation.

## Ethics Statement

The studies involving human participants were reviewed and approved by the Institutional Review Boards of China-Japan Friendship Hospital (approval number: 2015-23). The patients/participants provided their written informed consent to participate in this study. Written informed consent was obtained from the individual(s) for the publication of any potentially identifiable images or data included in this article.

## Author Contributions

HY acquired, analyzed, and interpreted the eASL data, and drafted the manuscript. YL conducted the statistical analysis. LZ and ZL acquired the clinical information. ZY assisted with the ASL data analysis. YF analyzed and interpreted the head and neck carotid Doppler ultrasound data. WG and YC acquired the MRA data. SX designed the study and revised the manuscript. All authors contributed to the article and approved the submitted version.

## Conflict of Interest

ZY was an employee of AnImageTech, who assisted with the ASL data analysis. The remaining authors declare that the research was conducted in the absence of any commercial or financial relationships that could be construed as a potential conflict of interest.

## Publisher’s Note

All claims expressed in this article are solely those of the authors and do not necessarily represent those of their affiliated organizations, or those of the publisher, the editors and the reviewers. Any product that may be evaluated in this article, or claim that may be made by its manufacturer, is not guaranteed or endorsed by the publisher.
